# Assessment of Knowledge, Attitudes, and Behaviors Toward Others’ Mental Disorders to Improve Mental Health First Aid: Cross-Sectional Study in a French Workplace

**DOI:** 10.2196/93207

**Published:** 2026-08-21

**Authors:** Matthieu Lebrat, Florence Carrouel, Benjamin du Sartz de Vigneulles, Michel Lamure, Corélie Salque, Roger Salamon, Catherine Massoubre, Nicolas Franck, Claude Dussart

**Affiliations:** 1 P2S UR4129, Université Lyon 1 Lyon Cedex 08 France; 2 Pharmacie et Stérilisation Centrales, Hospices Civils de Lyon Lyon, Rhône-Alpes France; 3 Institute of Public Health, Epidemiology and Development (ISPED), Inserm U1219, Université de Bordeaux Bordeaux, Nouvelle-Aquitaine France; 4 Research Unit EA7423, Université Jean Monnet Saint-Étienne, Auvergne-Rhône-Alpes France; 5 Department of Psychiatry, Université Jean Monnet Saint-Etienne, Auvergne-Rhône-Alpes France; 6 Centre Ressource de Réhabilitation psychosociale, Pôle Centre Rive Gauche, Centre Hospitalier Le Vinatier Bron, Auvergne-Rhône-Alpes France; 7 UMR 5229 CNRS, Centre Hospitalier Le Vinatier Bron, Auvergne-Rhône-Alpes France

**Keywords:** public health, health promotion, mental health, Mental Health First Aid, knowledge, attitudes, and behaviors, health literacy, civil servants, workplace prevention, stigma

## Abstract

**Background:**

Mental distress represents a major public health challenge worldwide, affecting work ability, productivity, and social functioning. Public-sector employees face high psychosocial demands, but data on their mental health literacy and supportive behaviors remain scarce. Mental Health First Aid (MHFA) aims to improve knowledge, attitudes, and behaviors (KAB) toward psychological distress among nonspecialists, but evidence among civil servants and on skill retention over time is limited.

**Objective:**

This study aimed (1) to determine KAB related to psychological distress among French civil servants, (2) to examine associations between KAB scores and MHFA training exposure, and (3) to explore gradients according to time since training as an indirect indicator of potential retention of MHFA-related competencies.

**Methods:**

A nationwide cross-sectional survey was conducted in November 2025 among French civil servants. Participants completed an online questionnaire adapted from a published instrument. The 3 dimension scores were normalized to a 0-40 scale, yielding a total KAB score of 0-120, categorized as inadequate (0-70), marginal (71-88), or adequate (89-120). Multivariable linear regression models identified factors associated with KAB outcomes, adjusting for sociodemographic and professional variables.

**Results:**

Among 338,560 eligible civil servants invited by email, 6526 (1.93%) initiated the questionnaire, and 4679 (1.38% of those invited) fully completed it and were included in the analysis. Participants were predominantly women (3022/4679, 64.59%), aged 40-69 years (4317/4679, 92.26%), and belonged to professional category A (2207/4679, 47.17%). The mean total KAB score was 82.88 (SD 19.14; 95% CI 82.33-83.43), corresponding to a marginal level. Mean subscores were 31.31 (SD 5.26; 95% CI 31.16-31.46) for knowledge, 29.72 (SD 11.52; 95% CI 29.39-30.05) for attitudes, and 21.86 (SD 7.96; 95% CI 21.63-22.09) for behaviors. MHFA-trained participants (545/4679, 11.65%) obtained significantly higher total KAB scores than untrained participants (mean 95.56, SD 14.17; 95% CI 94.37-96.75 vs mean 81.21, SD 19.09; 95% CI 80.63-81.79; *P*<.001), with higher scores for knowledge (mean 33.31, SD 5.26 vs mean 31.04, SD 5.35), attitudes (mean 34.35, SD 8.81 vs mean 29.10, SD 11.70), and behaviors (mean 27.87, SD 6.13 vs mean 21.06, SD 7.84; all *P*<.001). Among MHFA-trained participants, scores decreased over time (<6 months, 6-12 months, >12 months; *P*<.001), but remained higher than in untrained individuals even beyond 12 months after training. In adjusted models, MHFA training was strongly associated with higher KAB scores (β=14.63 for <6 months; β=13.61 for 6-12 months; β=10.33 for >12 months; all *P*<.001). Lower scores were observed in professional categories B and C and among men.

**Conclusions:**

Mental health literacy and supportive behaviors among French civil servants remain heterogeneous and, on average, marginal. MHFA training is associated with substantially higher KAB scores, even beyond 12 months, supporting its role as a public health literacy intervention in occupational settings. Declining scores over time supports reinforcement strategies to sustain competencies.

## Introduction

Mental health disorders—defined in the *International Classification of Diseases, 11th Revision* as clinically significant disturbances in cognition, emotional regulation, or behavior associated with distress or impairment in personal, social, educational, or occupational functioning—represent one of the leading public health challenges worldwide [1]. Nearly 1 in 7 people globally, more than a billion individuals, are living with a mental disorder, with depressive and anxiety disorders being the most prevalent. Mental disorders are the leading cause of suicide [2], and also the leading cause of years lived with disability globally, accounting for 1 in 6 years lived with disability [3,4]. The COVID‑19 pandemic further intensified this burden, leading to an estimated 27.6% increase in major depressive disorders and a 25.6% increase in anxiety disorders worldwide [5].

Against this global background, similar trends have been observed in France, where data from 2017 to 2021 indicate a clear increase in major depressive episodes, by roughly 3 to 4 percentage points, as well as a high prevalence of anxiety disorders, which affects nearly one-fifth of adults depending on the subgroup considered (men or women) [6]. These findings are consistent with international observations [7] and point to a significant deterioration in the mental health of the French population.

Mental health disorders contribute substantially to disability, reduced social participation, and impaired occupational functioning. Psychosocial distress also plays a significant role in long-term sickness absence, with substantial indirect costs (particularly productivity losses linked to difficulties in maintaining employment), which are often more costly than direct health care expenditures [3]. Thus, the burden of mental disorders and their societal impacts has led to the designation of mental health as France’s “National Cause” for 2025 and 2026, reflecting its strategic importance for societal sustainability [8].

More specifically, public sector employees are affected. In 2023, 19% of public-sector employees presented a risk of depressive symptoms [9], which is concerning, given their essential societal role and frequent exposure to emotionally demanding or high-responsibility situations [10,11]. Epidemiological studies have shown that workload, low decision latitude, and imbalance between effort and reward significantly contribute to the onset of common mental disorders [12]. Recent international guidelines from the World Health Organization (WHO) and the International Labour Organization emphasize the responsibility of governments, both as public employers and regulators, to prevent work-related mental disorders and support employees experiencing psychological difficulties [13,14]. Explicit consideration of the mental health of public-sector workers is thus essential for service quality, sustainability of public systems, and collective resilience. In this perspective, strengthening the capacity of civil servants to identify, understand, and respond appropriately to psychological distress emerges as a strategic priority.

Mental Health First Aid (MHFA) first emerged in Australia as a relevant resource. It aims to improve mental health literacy and reduce stigma among the general public. It also equips people with the skills to encourage those with mental issues to seek professional help. In France, the MHFA program, named “Premiers Secours en Santé Mentale,” has expanded rapidly since 2018, with more than 120,000 trained “first aiders” and over 1400 certified instructors as of May 2024 [15]. This rapid dissemination reflects strong institutional interest in equipping nonclinicians with the skills needed to recognize early signs of psychological distress and provide timely support. In 2022, a ministerial directive requested the establishment of MHFA within public employers [16]. Although several French studies [17,18] have highlighted the promising effects of MHFA training on trainees, these findings emphasize the need for additional analysis to further optimize the program [19]. Indeed, existing evidence is primarily based on student populations and has not specifically examined long-term effects. Consequently, it remains unclear whether similar benefits can be observed beyond the short term and in other populations such as civil servants. This group differs substantially in age and professional constraints, while their predefined socioprofessional classification offers a methodological asset for analyzing socioprofessional gradients.

Within the framework of improving mental health support in the workplace, particularly in the civil service, it is therefore essential to assess whether employees are able to recognize signs of psychological distress in their colleagues and respond appropriately. This requires a structured approach based on the knowledge, attitudes, and behaviors (KAB) model, which captures how individuals perceive, evaluate, and act upon a given situation. This can shed light on levels of mental health literacy. Indeed, KAB allows measurement of (1) knowledge of symptoms, risk factors, and help‑seeking pathways; (2) attitudes regarding stigma and self‑efficacy; and (3) supportive behaviors, including intent to help and referral practices. The KAB framework originates from health behavior and public health research and assumes that knowledge influences attitudes, which in turn may shape behaviors. Although these relationships are not necessarily linear, KAB models provide a useful framework for assessing health literacy and informing prevention strategies [20-22]. To date, despite the expansion of MHFA, no study has evaluated mental health KAB among civil servants in France, compared MHFA trained and untrained agents, or examined the persistence of skills over time in this context. Addressing this gap is crucial for informing national strategies aimed at strengthening mental health–related competencies within the civil service.

This study aimed (1) to determine KAB related to psychological distress among French civil servants, (2) to examine associations between KAB scores and MHFA training exposure, and (3) to explore gradients according to time since training as an indirect indicator of potential retention of MHFA-related competencies.

## Methods

### Study Design

This study adopted a descriptive, cross-sectional, quantitative design to examine KAB regarding mental disorders in others. Such a design is well suited to KAB surveys, particularly when data are scarce and baseline information is needed before developing interventions [21,23]. This study was performed in accordance with the CROSS (Checklist for Reporting Of Survey Studies; Multimedia Appendix 1) [24].

### Study Setting

This cross-sectional KAB survey was conducted in France from November 13-27, 2025, among active French civil servants who had a digital personal account on the national health insurance website and had agreed to receive information by email. Within the French civil service, positions are organized into 3 main professional categories: A, B, and C, which represent different corps of professions characterized by varying levels of responsibility, qualification, remuneration, and recruitment criteria. These categories structure the civil service hierarchy, with specific access conditions and baseline remuneration associated with each [9].

### Participants

#### Eligibility Criteria

To be included, participants had to (1) be at least 18 years of age, (2) be active public service employees, (3) have agreed to receive information at their email address, and (4) consent to complete the online questionnaire.

Retired individuals and nonpermanent civil servants (ie, public sector contract employees not belonging to public service category A, B, or C) were excluded from the study.

#### Recruitment

The recruitment frame consisted of all French civil servants who had a personal digital account within the Union Régime Obligatoire Prévention Santé system and had previously agreed to receive email communications. A total of 338,560 individuals met these criteria and received the study invitation.

No sampling, stratification, or additional selection procedures were applied because the invitation was sent to the entire eligible recruitment frame. The invitation email and study information letter were distributed to the entire eligible recruitment frame, followed by 1 reminder sent 1 week later to the same distribution list.

Participation was entirely voluntary. Individuals who provided informed consent, met the eligibility criteria, and initiated the online questionnaire were included in the study. Participants who completed all items required for KAB score calculation were included in the final analyses.

### Questionnaire

The French questionnaire developed and used by Llopis et al [17] to assess mental health–related knowledge, attitudes, and practices (KAP) among French university students who received MHFA training was adapted for the study population. The questionnaire was not originally developed as a psychometric instrument, and no formal psychometric validation was performed in this study. Specifically, age and date of MHFA training were collected using predefined categories, job position and education level items were adapted for relevance to French public service classifications, and some items were removed to reduce questionnaire length. In total, 5 questions were adapted for this study population, 1 question was shortened, and the other 32 items were retained in their original form. No new items were created. The wording of the questions was kept as original.

It comprised 38 closed-ended questions and covered the following domains: (1) sociodemographic data (age collected in ranges and job position collected according to French public service categories A, B, and C) and information on MHFA training (including motivations and time since training: <6, 6-12, and >12 months)—7 questions, (2) mental health knowledge—10 questions, (3) attitudes toward people with mental health disorders—4 questions, and (4) behaviors toward individuals experiencing psychological difficulties—17 questions. Among these questions, 5 were specifically reserved for MHFA trainees. The survey required approximately 10 minutes to complete. The original French version and an English translation of the questionnaire are provided in Multimedia Appendices 2 and 3.

### Scoring System

From the collected responses, new variables were created: knowledge score related to mental health: sum of items 8 to 17 (1 point per correct response and 0 points per incorrect response or “do not know”). Attitude score toward people with psychological distress: sum of items 18 to 21 (1 point per correct response and 0 points per incorrect response or “do not know”). Behavior score toward individuals experiencing psychological distress, to assess the level of consistency with the recommendations made during MHFA training: sum of items 22 to 26 and 31 to 38 (1 point per correct response and 0 points per incorrect response or “do not know.” Likert items scored 0-4). This scoring system was developed in accordance with the WHO KAP survey methodology [20], which recommends simplified and direction-oriented coding approaches for population-based assessments of health-related KAB. The normalization and categorization procedures were adopted to facilitate interpretation and comparison of KAB dimensions at the population level. Equal weighting of items was intentionally adopted because the objective of the study was to generate descriptive population-level indicators rather than to develop a diagnostic or psychometric scale. No empirical or theoretical framework was available to justify differential weighting of individual items. Consequently, each item contributed equally to its corresponding KAB dimension, consistent with WHO KAP survey methodology and previous population-based KAP or KAB surveys.

Scores for each KAB dimension were normalized on a scale from 0 to 40, with total KAB score ranging from 0 to 120. Because the 3 KAB dimensions were based on different numbers of items and score ranges, raw scores were transformed to a common 0-40 scale using a linear normalization procedure according to the following formula:

Normalized score=(observed score/maximum possible score)×40 **(1)**

This transformation allowed direct comparison of the 3 dimensions while preserving the relative position of participants within each dimension. The total KAB score corresponded to the sum of the 3 normalized dimension scores and therefore ranged from 0 to 120. Details of the raw and normalized score ranges are provided in Multimedia Appendix 4.

Categories were set as follows: inadequate (0-70), marginal (71-88), and adequate (89-120) based on previous questionnaires [23]. These categories were intended to facilitate interpretation of population-level KAB results and should not be considered clinically validated thresholds.

### Survey Platform

The questionnaire was hosted online using LimeSurvey (version 6.16.5; LimeSurvey GmbH) on a dedicated server with 2048 bitset Secure Sockets Layer/Transport Layer Security encryption and without collecting IP addresses. Measures were taken to ensure secure, anonymous participation, cookies to block duplicate submissions, and unique tokens for log-in. Respondents could pause and resume the survey as needed.

### Study Outcomes

The primary outcome was to determine KAB of civil servants when addressing psychological distress in others.

The secondary outcomes were to examine associations between KAB scores and MHFA training exposure and to explore gradients according to time since training as an indirect indicator of potential retention of MHFA-related competencies. Accordingly, analyses comparing MHFA-trained and untrained participants, as well as analyses according to time since training (<6, 6-12, and >12 months), were predefined secondary exploratory analyses.

### Statistical Analysis

#### Sample Size Calculation

Based on an eligible population of 338,560 civil servants who received the email invitation, corresponding to the entire accessible Union Régime Obligatoire Prévention Santé recruitment frame, a minimum of 1064 completed questionnaires was calculated to ensure a 95% confidence level and a ±3 percentage-point margin of error for proportion estimates (finite population correction applied). This calculation was derived from the standard formula for estimating a proportion, assuming a conservative prevalence of 50% to maximize variance:

*n*₀=*Z*^2^×*p*(1–*p*)/*e*^2^**(2)**

where *Z*=1.96 (95% confidence level), *p*=0.50 (maximum variance assumption), and *e*=0.03 (margin of error). The resulting sample size was then adjusted using the finite population correction:

*n*=*n*₀/[1+(*n*₀–1)/*N*] **(3)**

where *N*=338,560. The final minimum sample size was estimated at 1064 completed questionnaires.

#### Data Analysis

Data were analyzed using R (R Foundation for Statistical Computing) via RStudio. Descriptive statistics were computed using standard R packages, and forest plots were generated using the *ggplot2* package. Descriptive statistics (counts and percentages) were used for categorical sociodemographic variables and general information related to MHFA training. Means and SDs were reported for continuous KAB score variables.

The sample was divided into 2 groups according to MHFA training status (yes vs no), and KAB scores were compared using 1-way ANOVA. Among participants who received MHFA training, 3 categories were defined based on time since training, and KAB scores were compared across these groups using 1-way ANOVA. Prior to conducting ANOVA analyses, normality was assessed using the Shapiro-Wilk test and visual inspection of quantile-quantile plots. Although the formal Shapiro-Wilk test indicated departures from normality, visual inspection suggested only minor deviations, and parametric analyses were considered appropriate, given the large sample size and the robustness of ANOVA to modest departures from normality. Homogeneity of variances was assessed using the Levene test. When the Levene test indicated heterogeneity of variances, corresponding nonparametric tests (Wilcoxon rank sum test with continuity correction or Kruskal-Wallis rank sum test, as appropriate) were performed as sensitivity analyses and yielded the same conclusions regarding statistical significance. Moreover, because comparisons according to MHFA training status and time since training were predefined secondary analyses addressing specific study objectives rather than exploratory post hoc analyses, no formal adjustment for multiple comparisons was applied. Achieved statistical power was estimated for the predefined subgroup analyses according to time since MHFA training.

All complete questionnaires were included in descriptive and inferential analyses, as complete KAB score calculation required complete responses for the corresponding items. To evaluate the potential for selection bias related to missing data, respondents with complete questionnaires were compared with participants who did not complete the questionnaire but who had provided at least the sociodemographic information required for comparison analyses (Multimedia Appendix 5). Because KAB scores were directly derived from questionnaire responses, imputation of missing outcome items would have required strong assumptions regarding unobserved responses. Consequently, analyses were restricted to complete questionnaires, and potential selection bias was evaluated through comparison of complete and incomplete respondents. Inverse probability weighting was not performed because comparisons between complete and incomplete respondents showed only negligible-to-small differences across the available baseline characteristics and no difference in MHFA training status, the primary exposure variable. In addition, only a limited set of baseline variables was available for incomplete respondents, preventing construction of a robust response-propensity model. Determinants of KAB scores were examined using multivariable linear regression models. Covariates included job position, level of education, age, gender, MHFA training status, and time since training. All covariates were modeled as categorical variables according to the categories in which they were collected. For each variable, one category was used as the reference category in the regression models. Models were estimated by using generalized linear modeling procedures in R.

Regression coefficients (β) were reported with 95% CIs and associated *P* values. A 2-sided *P* value <.05 was considered statistically significant.

### Ethical Considerations

The study was conducted in accordance with the Declaration of Helsinki and approved by the research ethics committee of the Vinatier Hospital, Lyon, France (CEREVI/2025/45; July 7, 2025). Before accessing the questionnaire, all participants received study information electronically and provided informed consent to participate. Participation was entirely voluntary, and participants could discontinue the questionnaire at any time without consequence. To protect privacy and confidentiality, the questionnaire was administered anonymously through a secure online platform. No IP addresses were collected, and all data were analyzed in aggregated form only. Participants did not receive any financial compensation or incentive for participation.

## Results

### Characteristics of the Participants

**Figure 1:**
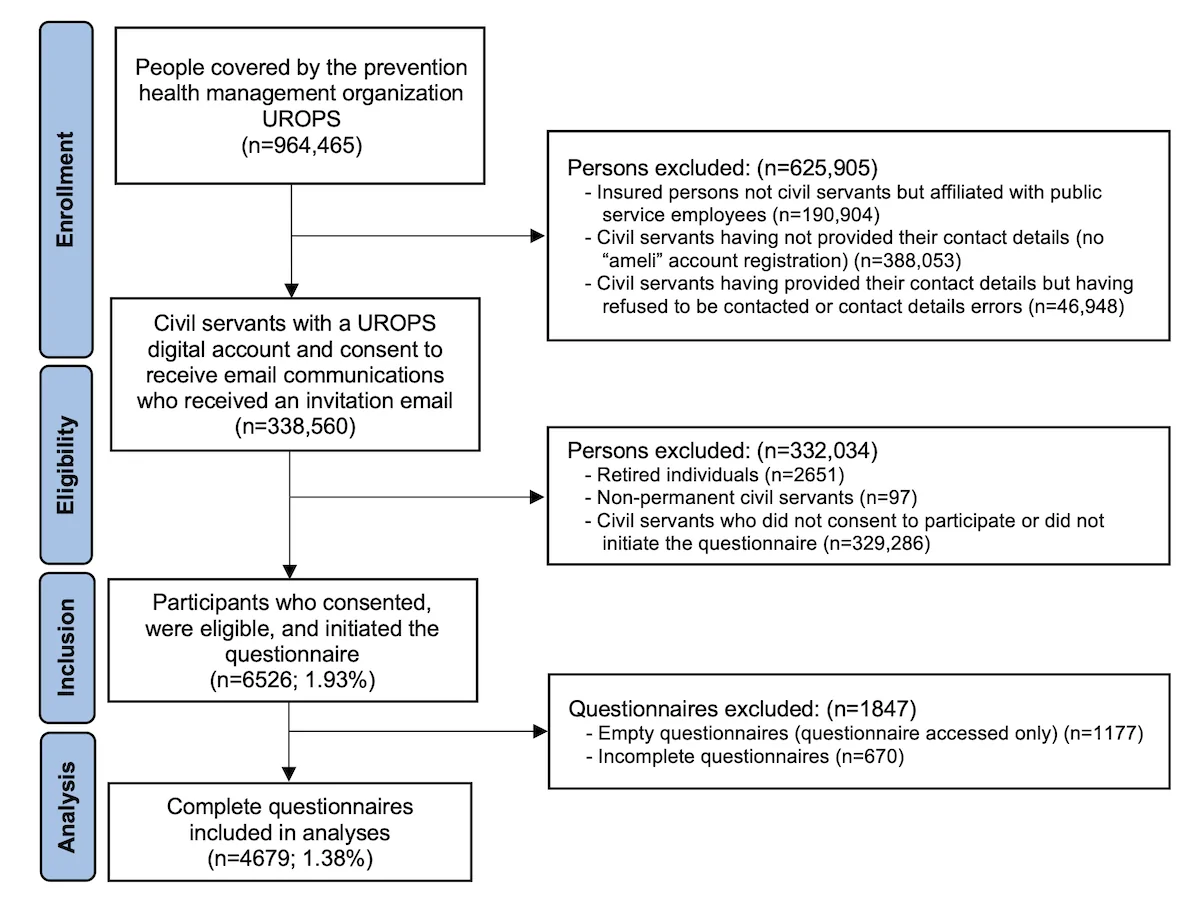
Flowchart of the study. UROPS: Union Régime Obligatoire Prévention Santé.

Figure 1 shows the flowchart of the study. A total of 338,560 civil servants received the study invitation. Among them, 6526 (1.93%) individuals provided consent, were eligible, and initiated the questionnaire. Of these participants, 4679 (1.38% of invited individuals) completed all items required for KAB score calculation and were included in the final analyses. This sample size, well above the calculated minimum, ensured high statistical precision, whereas the voluntary recruitment process may limit full population representativeness.

Table 1 shows the sociodemographic characteristics and information related to MHFA training of the participants. Most participants were women (3022/4679, 64.59%), aged between 40 and 69 years (4317/4679, 92.26%), and belonged to the A professional category (2207/4679, 47.17%). Participants with an undergraduate degree (first-cycle university degree and second-cycle university degree) were predominant (2220/4679, 47.45%). Most participants had never received training in mental health (3652/4679, 78.05%) and had not had the opportunity to follow an MHFA training (4134/4679, 88.35%).

The distribution of questionnaire breakoff according to the last completed questionnaire item is presented in Multimedia Appendix 6. Characteristics of complete and incomplete respondents are presented in Multimedia Appendix 5. Compared with respondents who completed the questionnaire, those with incomplete questionnaires differed slightly in age, gender, professional category, and educational level (all *P*<.001). However, effect sizes were negligible to small (Cramer *V*=0.06-0.11), suggesting limited practical differences between groups. No significant differences were observed regarding previous mental health training (*P*=.20) or MHFA training status (*P*=.75).

**Table 1:** Sociodemographic characteristics and information related to Mental Health First Aid (MHFA) training of the study population (N=4679).

Variables			Participants, n (%)
Age range (years)			
	18-29	59 (1.26)	
	30-39	303 (6.48)	
	40-49	1075 (22.97)	
	50-59	1924 (41.12)	
	60-69	1318 (28.17)	
Gender			
	Woman	3022 (64.59)	
	Man	1650 (35.26)	
	Other	7 (0.15)	
Professional category			
	A	2207 (47.17)	
	B	1777 (37.98)	
	C	695 (14.85)	
Level of education			
	No diploma	39 (0.83)	
	Below high school diploma	433 (9.25)	
	High school diploma^a^	959 (20.50)	
	First-cycle university degree^b^	878 (18.76)	
	Second-cycle university degree^c^	1342 (26.68)	
	Third-cycle university degree^d^	1028 (21.97)	
Previous mental health training			
	Yes	1027 (21.95)	
	No or do not know	3652 (78.05)	
Previous MHFA training			
	Yes, <6 months ago	216 (4.62)	
	Yes, 6-12 months ago	117 (2.50)	
	Yes, >12 months ago	212 (4.53)	
	No	4134 (88.35)	

^a^French baccalaureate.

^b^Baccalaureate+2 years.

^c^Baccalaureate+3 years.

^d^Baccalaureate+5 years and more.

### KAB Scores in the Civil Servant Population in France

Table 2 presents descriptive statistics of KAB scores. In the overall civil servant population (N=4679), the mean total KAB score was 82.88 (SD 19.14; 95% CI 82.33-83.43), based on a normalized scale theoretically ranging from 0 to 120 points, corresponding to a “marginal” level according to the predefined scoring thresholds.

The mean scores for the 3 subdimensions—knowledge, attitudes, and behaviors—were 31.31 (SD 5.26; 95% CI 31.16-31.46), 29.72 (SD 11.52; 95% CI 29.39-30.05), and 21.86 (SD 7.96; 95% CI 21.63-22.09), respectively, each based on a normalized scale theoretically ranging from 0 to 40 points.

KAB scores differed according to MHFA training status and, among trained participants, by time since training (Table 2). Participants who had received MHFA training had a significantly higher mean KAB score than those without training (mean 95.56, SD 14.17; 95% CI 94.37-96.75 vs mean 81.21, SD 19.09; 95% CI 80.63-81.79; *P*<.001). Similar patterns were observed for each KAB dimension. Trained participants showed higher mean scores for knowledge (mean 33.31, SD 3.96; 95% CI 32.98-33.64 vs mean 31.04, SD 5.35; 95% CI 30.88-31.20; *P*<.001), attitudes (mean 34.35, SD 8.81; 95% CI 33.61-35.09 vs mean 29.10, SD 11.70; 95% CI 28.74-29.46; *P*<.001), and behaviors (mean 27.87, SD 6.13; 95% CI 27.36-28.38 vs mean 21.06, SD 7.84; 95% CI 20.82-21.30; *P*<.001).

In addition to statistically significant differences, the comparison between MHFA-trained and untrained participants showed small-to-moderate effect sizes for knowledge (Cohen *d*=0.44) and attitudes (*d*=0.46), a large effect size for behaviors (*d*=0.89), and a moderate-to-large effect size for the overall KAB score (*d*=0.77; Multimedia Appendix 7).

Among participants who had completed MHFA training, a decreasing gradient of mean scores was observed with increasing time since training (<6, 6-12, and >12 months) for the total KAB score (*P*<.001) as well as for knowledge, attitudes, and behaviors scores over time (*P*<.001, *P*=.04, and *P*<.001, respectively). Results of the post-hoc power calculations for these subgroup analyses are presented in Multimedia Appendix 8.

**Table 2:** Descriptive statistics and univariable comparisons of knowledge, attitudes, and behaviors (KAB) scores by Mental Health First Aid (MHFA) training status and time since training.

MHFA training status	All (N=4679), mean (SD; 95% CI)	No (n=4134), mean (SD; 95% CI)	Yes (n=545), mean (SD; 95% CI)	Yes (<6 months; n=216), mean (SD; 95% CI)	Yes (6-12 months; n=117), mean (SD; 95% CI)	Yes (>12 months; n=212), mean (SD; 95% CI)	*P* value (yes or no)^a^	Cohen *d*	*P* value (<6 or 6-12 or >12 months)^b^
Knowledge score^c^	31.31 (5.26; 31.16-31.46)	31.04 (5.35; 30.88-31.20)	33.31 (3.96; 32.98-33.64)	34.07 (3.58; 33.59-34.55)	33.50 (3.76; 32.82-34.18)	32.42 (4.27; 31.85-32.99)	<.001	0.44	<.001
Attitude score^c^	29.72 (11.52; 29.39-30.05)	29.10 (11.70; 28.74-29.46)	34.35 (8.81; 33.61-35.09)	35.32 (7.53; 34.32-36.32)	34.10 (9.48; 32.38-35.82)	33.58 (9.56; 32.29-34.87)	<.001	0.46	.04
Behavior score^c^	21.86 (7.96; 21.63-22.09)	21.06 (7.84; 20.82-21.30)	27.87 (6.13; 27.36-28.38)	28.7 (5.47; 27.97-29.43)	28.49 (5.38; 27.52-29.46)	26.66 (6.93; 25.73-27.59)	<.001	0.89	<.001
KAB score^d^	82.88 (19.14; 82.33-83.43)	81.21 (19.09; 80.63-81.79)	95.56 (14.17; 94.37-96.75)	98.12 (11.74; 96.55-99.69)	96.09 (14.33; 93.49-98.69)	92.67 (15.79; 90.54-94.80)	<.001	0.77	<.001

^a^One-way ANOVA comparing MHFA-trained and untrained participants. When the Levene test indicated heterogeneity of variances, results were confirmed using the Wilcoxon rank sum test with continuity correction.

^b^One-way ANOVA comparing KAB scores according to time since MHFA training. When the Levene test indicated heterogeneity of variances, results were confirmed using the Kruskal-Wallis rank sum test.

^c^Score between 0 and 40.

^d^Score between 0 and 120.

### Identification of Determinants Associated With KAB Scores

To examine factors associated with KAB scores while accounting for potential confounders, multivariable linear regression models were fitted. These analyses aimed to identify variables statistically associated with KAB outcomes, rather than to infer causal effects.

#### Total KAB Score

**Figure 2:**
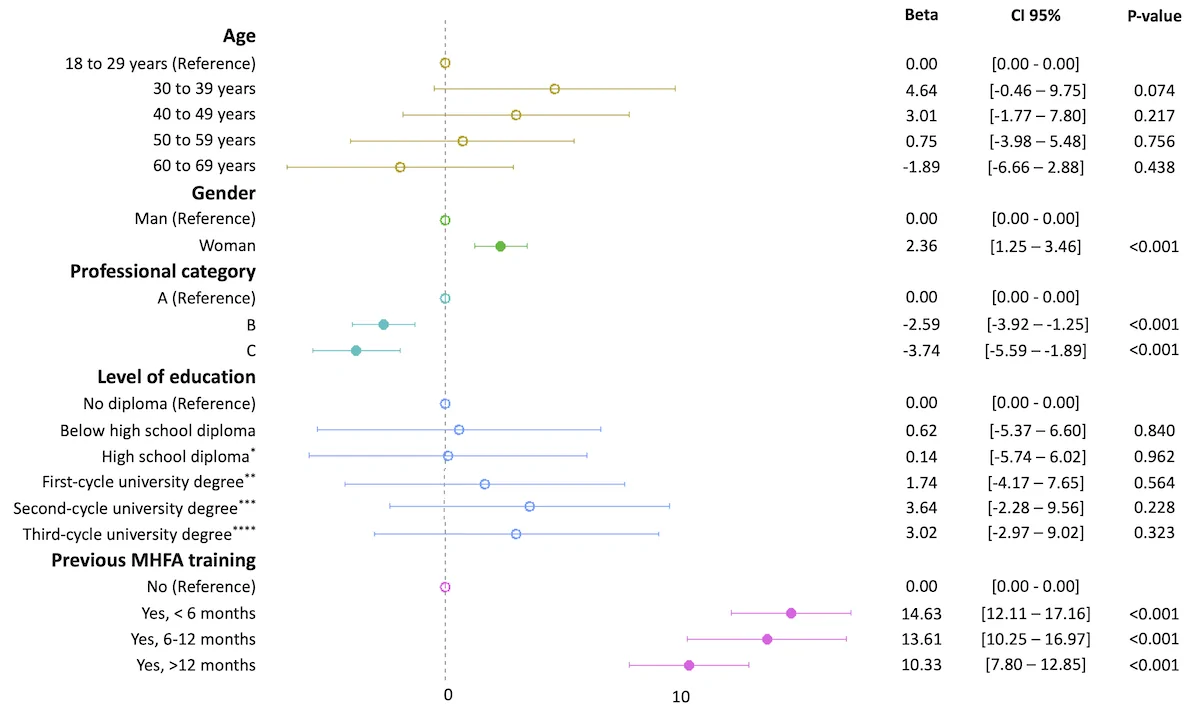
Forest plot representing the determinants of the KAB score regarding psychological distress in others. Filled circles indicate statistical significance (P≤.05), while empty circles indicate nonsignificance (*P*>.05). Reference categories are specified in parentheses next to the variable labels. KAB: knowledge, attitudes, and behaviors; MHFA: Mental Health First Aid. *French baccalaureate, **Baccalaureate+2 years, ***Baccalaureate+3 years, ****Baccalaureate+5 years and more.

As shown in Figure 2, previous MHFA training was strongly associated with higher total KAB scores. Compared with untrained participants, mean KAB scores were higher among those trained: less than 6 months ago (β=14.63, 95% CI 12.11-17.16; *P*<.001), 6-12 months ago (β=13.61, 95% CI 10.25-16.97; *P*<.001), and more than 12 months ago (β=10.33, 95% CI 7.80-12.85; *P*<.001).

Women had higher total KAB scores than men (β=2.36, 95% CI 1.25-3.46; *P*<.001). In contrast, participants in professional categories B and C had lower KAB scores compared with those in category A, with mean decreases of 2.59 (95% CI –3.92 to –1.25) points and 3.74 (95% CI –5.59 to –1.89) points, respectively (all *P*<.001).

#### Knowledge Score

**Figure 3:**
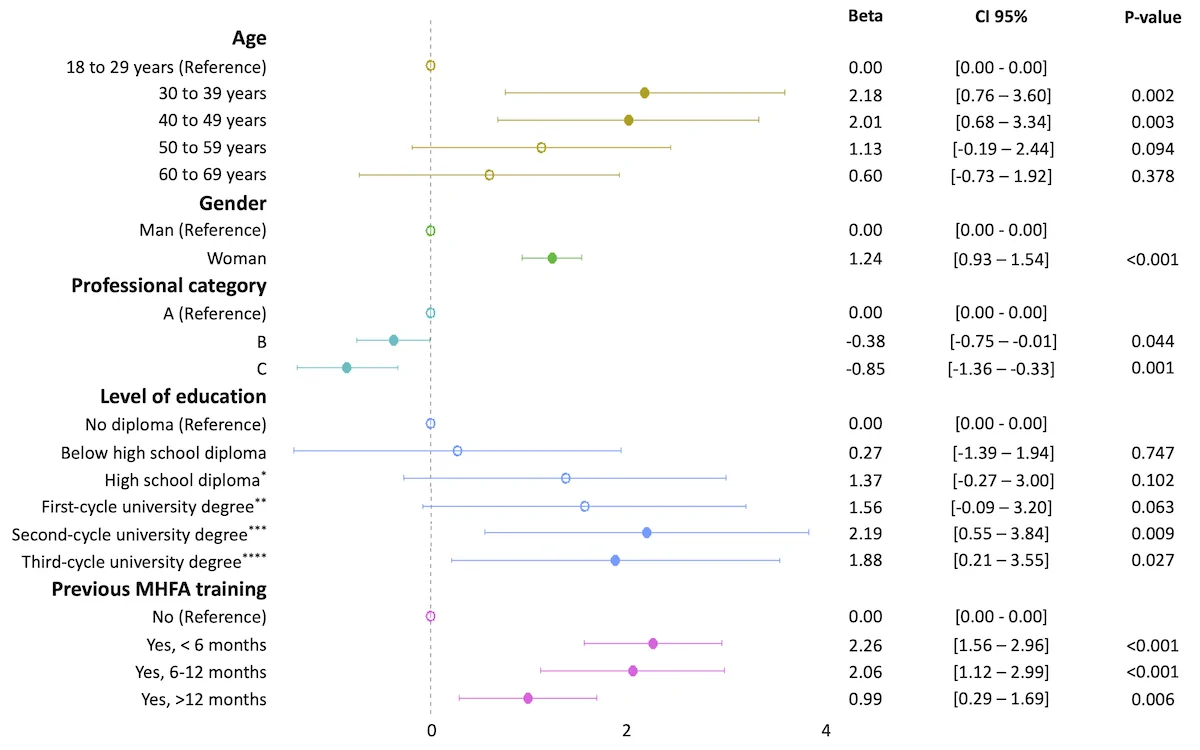
Forest plot representing the determinants of the knowledge score regarding psychological distress in others. Filled circles indicate statistical significance (P≤.05), while empty circles indicate nonsignificance (*P*>.05). Reference categories are specified in parentheses next to the variable labels. MHFA: Mental Health First Aid. *French baccalaureate, **Baccalaureate+2 years, ***Baccalaureate+3 years, ****Baccalaureate+5 years and more.

As shown in Figure 3, higher knowledge scores were associated with MHFA training status, women gender, and higher professional category. Participants aged 30-39 and 40-49 years had higher knowledge scores than those aged 18-29 years (β=2.18, 95% CI 0.76-3.60; *P*=.002 and β=2.01, 95% CI 0.68-3.34; *P*=.003, respectively). Higher knowledge scores were also observed among participants with an undergraduate degree or a postgraduate or graduate degree compared with those without a diploma (β=2.19, 95% CI 0.55-3.84; *P*=.009 and β=1.88, 95% CI 0.21-3.55; *P*=.03, respectively).

#### Attitude Score

**Figure 4:**
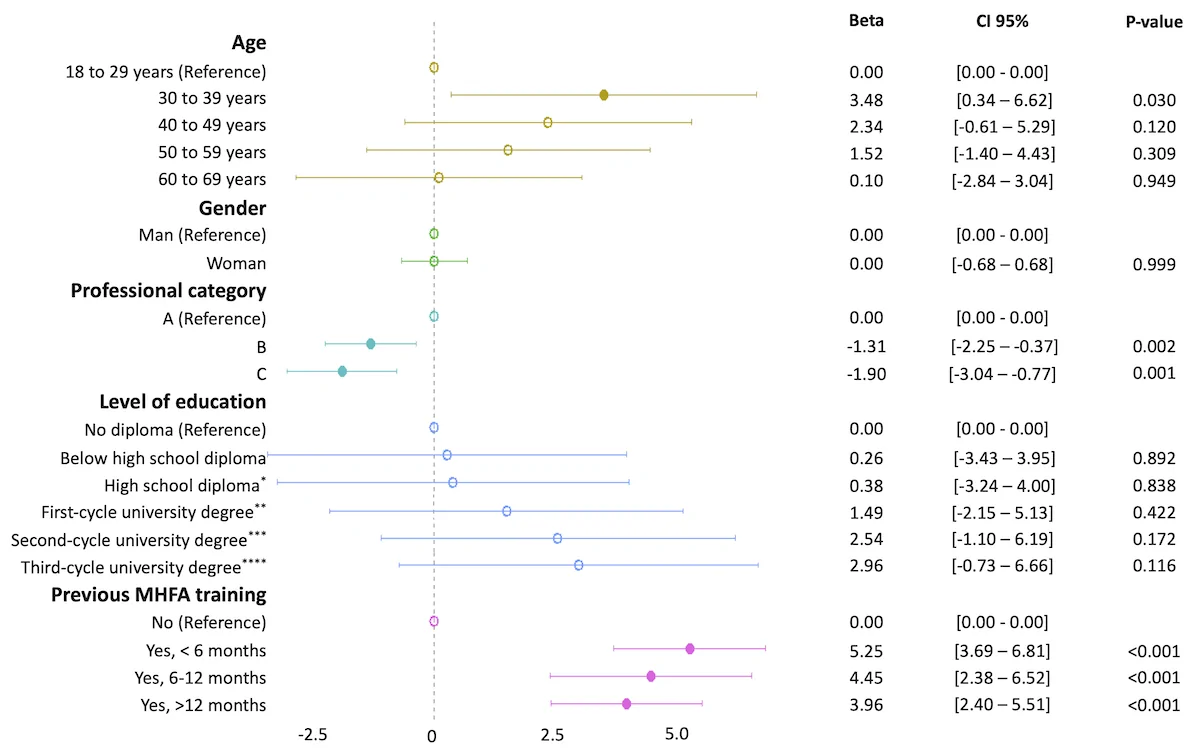
Forest plot representing the determinants of the attitude score regarding psychological distress in others. Filled circles indicate statistical significance (P≤.05), while empty circles indicate nonsignificance (*P*>.05). Reference categories are specified in parentheses next to the variable labels. MHFA: Mental Health First Aid. *French baccalaureate, **Baccalaureate+2 years, ***Baccalaureate+3 years, ****Baccalaureate+5 years and more.

As shown in Figure 4, attitude scores were positively associated with MHFA training across all time categories. Participants trained less than 6 months ago had the highest adjusted attitude scores, followed by those trained 6-12 months and more than 12 months ago.

Professional categories B and C were negatively associated with attitude scores compared with category A. Age, gender, and education level were not significantly associated with attitude scores in adjusted models, except participants aged 30-39 years who had significantly higher attitude scores than those aged 18-29 years (β=3.48, 95% CI 0.34-6.62; *P*=.03).

#### Behavior Score

**Figure 5:**
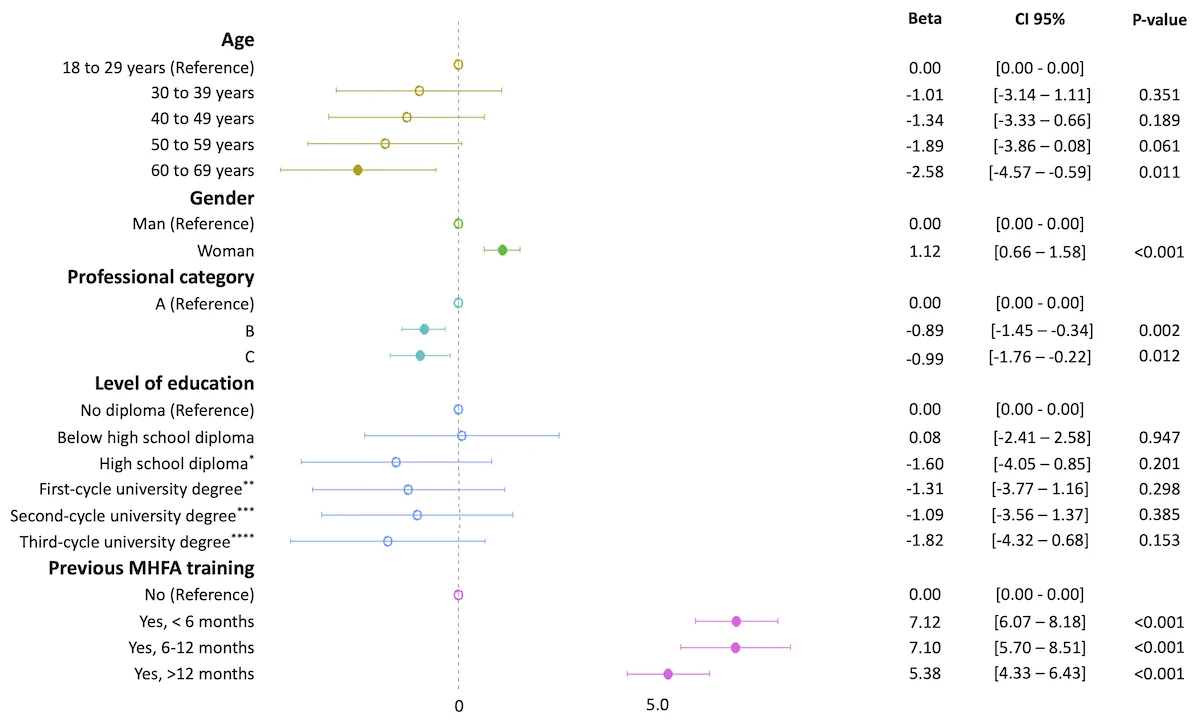
Forest plot representing the determinants of the behavior score regarding psychological distress in others. Filled circles indicate statistical significance (P≤.05), while empty circles indicate nonsignificance (*P*>.05). Reference categories are specified in parentheses next to the variable labels. MHFA: Mental Health First Aid. *French Baccalaureate, **Baccalaureate+2 years, ***Baccalaureate+3 years, ****Baccalaureate+5 years and more.

As shown in Figure 5, MHFA training status was strongly associated with higher behavior scores across all time categories. Woman gender was also associated with higher behavior scores, whereas increasing age and lower professional categories were associated with lower scores.

### Motivations, Knowledge Retention, and Application of MHFA Skills Among Trained Civil Servants

Table 3 describes the motivations for MHFA training, knowledge of ALGEE (Approach, Listen, Give support, Encourage professional help, Encourage other supports), and application in practice. Among the 545 participants who had completed MHFA training, the majority reported enrolling for reasons related to their professional or academic environment (384/545, 70.46%), while over half cited the desire to better support family members or friends (290/545, 53.21%). Additional motivations included a personal interest in mental health (228/545, 41.83%), the wish to better understand their own mental health (188/545, 34.50%), and civic engagement (193/545, 35.41%). Fewer participants indicated motivations related to the COVID-19 crisis (26/545, 4.77%) or the free nature of the training (33/545, 6.06%).

Assessment of core MHFA concepts showed high levels of retention of key components of the ALGEE action plan (a major component of MHFA training), with over 97% correctly identifying the importance of listening without judgment (531/545, 97.43%) and providing comfort and information (528/545, 96.88%). However, some participants still endorsed less appropriate responses, such as “encouraging the person to pull themselves together” (206/545, 37.80%) or “trying to solve their problems” (137/545, 25.14%).

Approximately one-third of trained participants (194/545, 35.60%) reported having used MHFA skills to help someone experiencing psychological distress. Among these, most had supported 1 to 4 individuals (145/545, 26.61%), while a smaller number reported helping 5 or more (49/545, 8.99%). Among those who had used their training, the majority (170/194, 87.63%) perceived it as helpful in supporting the person in distress.

**Table 3:** Motivations for Mental Health First Aid (MHFA) training, knowledge of ALGEE (Approach, Listen, Give support, Encourage professional help, Encourage other supports), and application in practice (n=545).

Variables			Participants, n (%)
Motivation for participation to MHFA training			
	To better support family members or friends	290 (53.21)	
	Related to and useful for my studies and/or profession	384 (70.46)	
	To improve my understanding of my own mental health	188 (34.50)	
	Due to my interest in the field of mental health	228 (41.83)	
	To engage in a civic action and act in the public interest	193 (35.41)	
	To address a mental health need arising from the COVID-19 crisis	26 (4.77)	
	Because this training was free	33 (6.06)	
	Other	22 (4.04)	
Level of knowledge of the ALGEE action plan among participants who had completed MHFA training			
	Trying to treat the person or find solutions to their problems^a^	408 (74.86)	
	Providing support and assessment in case of a crisis^b^	476 (87.34)	
	Providing comfort and information^b^	528 (96.88)	
	Listening actively and without judgment^b^	531 (97.43)	
	Encouraging the person to pull themselves together^a^	339 (62.20)	
Applied MHFA skills to help someone with a mental health problem			
	Yes	194 (35.60)	
	No or prefer not to say	351 (64.40)	
Number of people assisted (approximate)			
	0	351 (64.40)	
	1	58 (10.64)	
	2-4	87 (15.96)	
	5-7	22 (4.04)	
	8-10	8 (1.47)	
	>10	19 (3.49)	
Perceived usefulness of MHFA knowledge in helping the persons			
	Yes	170 (87.63)	
	No or do not know	24 (12.37)	

^a^Correct answer “No.”

^b^Correct answer “Yes.”

## Discussion

### Principal Findings

This large cross-sectional study provides the first comprehensive assessment of KAB related to psychological distress among French civil servants. The results reveal substantial heterogeneity within this population, with an overall mean KAB score of 82.88 of 120 (SD 19.14), classified as marginal (95% CI 82.33-83.43). From a public health perspective, it highlights that mental health competencies remain insufficiently consolidated in a workforce central to public service delivery and social functioning. While these observations provide valuable insight into mental health literacy within the French civil service, they are based primarily on self-reported measures and should therefore be interpreted with appropriate caution. In particular, reported use of MHFA-related skills and perceived usefulness of training reflect participants’ own perceptions and were not independently validated through recipient-level outcomes.

Mental health disorders are now among the leading causes of disability worldwide, with well-documented consequences for work ability, productivity, and long-term labor participation [3,25,26]. In this context, the marginal level observed highlights a gap between the growing burden of mental health needs and the collective capacity of institutions to recognize and respond to psychological distress. Addressing mental health in the workplace must thus go beyond clinical care and be integrated into public health strategies.

The strongest finding of this study is that MHFA-trained participants displayed substantially higher KAB scores than nontrained participants across all dimensions: knowledge (+2.3 points), attitudes (+5.3 points), and behaviors (+6.8 points). Trained participants had a total KAB score of 95.56 versus 81.21 for untrained individuals (*P*<.001). This interpretation is further supported by effect size estimates, which were moderate for the overall KAB score (Cohen *d*=0.77) and particularly large for the behavior dimension (*d*=0.89). However, given the cross-sectional design and the voluntary nature of participation in MHFA training, these differences should be interpreted as associations rather than evidence of a causal training effect. Importantly, participants who had completed MHFA training more than 12 months previously continued to display higher KAB scores than untrained individuals. Although lower scores were observed among participants reporting longer intervals since training, this pattern should be interpreted cautiously because time since training represents only an indirect indicator of retention and may also reflect differences in opportunities for skill application, workplace environment, refresher activities, or other unmeasured factors. Beyond these quantitative findings, responses from trained participants also provide insight into the motivations for attending MHFA training and the practical application of acquired skills. Most cited professional or academic reasons for attending MHFA (384/545, 70.46%), followed by the desire to support relatives or friends (290/545, 53.21%). These findings highlight the strategic relevance of MHFA training within the civil service and underscore participants’ recognition of the importance of mental health considerations in the workplace.

Core ALGEE principles were well retained—for example, 97.43% (531/545) recalled the importance of nonjudgmental listening—though some suboptimal reflexes persisted (eg, 206/545, 37.80% agreed with “pulling oneself together”). About 35.60% (194/545) had applied MHFA skills to help someone, most often 1 to 4 individuals, and 87.63% (170/194) found the training useful in doing so. These findings suggest that MHFA can be meaningfully translated into supportive behaviors.

Finally, the sample was predominantly women (3022/4679, 64.59%) and aged 50 years and older (3242/4679, 69.29%), aligning with broader public service demographics [9]. This should be considered when tailoring future training strategies to subgroups with differing needs or exposure.

### Comparison With Prior Work

The positive impact of MHFA training found in this study aligns with existing literature [27-31]. A 2018 meta-analysis by Morgan et al [27] showed consistent improvements in mental health knowledge, stigma reduction, and helping behaviors across diverse international contexts. Our findings extend these results to a civil service context—a group not yet well represented in MHFA research. Nevertheless, unlike the intervention studies cited earlier, this study was not designed to evaluate MHFA effectiveness. Participation in MHFA training was voluntary, and self-selection mechanisms may partly explain the higher KAB scores observed among trained participants. Consequently, our findings should be interpreted as evidence of an association between MHFA training exposure and KAB outcomes rather than proof of a causal effect.

French studies conducted among university students [17,18] as well as a randomized controlled trial in Switzerland [32] have shown significant posttraining improvements in knowledge, attitudes, and peer support capacities. Effects were particularly strong for proximal outcomes such as confidence and willingness to help. Our study confirms that these improvements are also observable in older, more professionally diverse cohorts, offering useful insights for public policy and workforce-based mental health strategies.

Beyond perceived improvements, this study documents the self-reported application of MHFA skills. Over one-third of trained participants reported supporting someone in psychological distress, with the vast majority considering their training helpful in doing so. These findings suggest that MHFA-related competencies may be actively mobilized in professional or social contexts. However, as highlighted in a systematic review by Forthal et al [33], the evidence on real-world outcomes remains mixed, and few studies have rigorously assessed whether actions taken by trainees are truly helpful or improve recipient outcomes. Consequently, this study cannot determine whether actions taken by trainees actually improved recipient outcomes, increased professional help-seeking, reduced psychological distress, or improved other recipient-level outcomes. Our findings therefore extend the MHFA literature by documenting self-reported application of MHFA skills, but independent validation of recipient outcomes remains an important gap in the literature. Further research using validated recipient-level outcomes is needed to establish the real-world impact of MHFA training.

Among MHFA-trained civil servants, a high level of retention of key elements of the ALGEE action plan was observed, particularly nonjudgmental listening (531/545, 97.43%) and providing comfort and information (528/545, 96.88%). These rates are in line with previous findings from student populations, where essential MHFA principles remained well retained months after training [17,32]. Nonetheless, some persistent misconceptions were noted—nearly 37.80% (206/545) of trained participants agreed with the idea of “encouraging the person to pull themselves together” [17], a response inconsistent with MHFA principles. This enduring influence of cultural or intuitive norms highlights the limits of single-session training and reinforces the need for ongoing reinforcement.

Our study provides exploratory observations regarding differences in KAB scores according to time since training. While lower scores were observed among participants reporting longer intervals since MHFA training, the cross-sectional design does not allow these differences to be interpreted as direct evidence of skill decay. Alternative explanations, including cohort effects, differences in training implementation, opportunities for skill application, workplace support, refresher training, or other unmeasured factors, cannot be excluded. This pattern is consistent with findings in health education and learning science, particularly the “spacing effect,” which shows that spaced learning supports long-term retention more effectively than one-time exposure [34,35]. These results argue for the inclusion of refresher sessions as part of long-term MHFA implementation strategies, especially in large and diverse workforces.

Interestingly, 11.65% (545/4679) of our sample reported having received MHFA training—substantially higher than national averages (estimated at ~0.2% of the general population) [15]. This suggests a possible selection bias, but may also reflect higher interest, access, or perceived relevance of MHFA among public sector employees. Notably, 70.46% (384/545) of trained participants cited professional or academic motivations, reinforcing the relevance of MHFA for emotionally demanding roles and public service functions.

Sociodemographic gradients in KAB scores were consistent with prior findings. Women scored higher across all dimensions, in line with studies showing greater mental health literacy and empathy among women respondents [36,37]. Participants in higher professional categories (category A) and with higher education levels also showed stronger outcomes, likely reflecting greater exposure to psychosocial issues or prior training. Similar patterns were reported in KAB-focused studies among civil servants, including in the context of long COVID [38], or oral health literacy [39], underlining the structural dimensions that shape prevention capacities.

Taken together, these findings confirm the relevance of MHFA in occupational settings and provide strong support for its application in workforces. However, to ensure sustainable impact, training should be combined with targeted reinforcement strategies and equitable access across all staff categories.

### Limitations

Several limitations should be acknowledged. First, the cross-sectional nature of the study precludes causal inference regarding MHFA training effectiveness. Because participation in MHFA training was voluntary, self-selection cannot be excluded, and trained participants may have differed from untrained participants in baseline mental health literacy, motivation, or interest in mental health. Consequently, the observed differences in KAB scores may partly reflect preexisting characteristics rather than training effects. Although multivariable analyses adjusted for several sociodemographic variables, residual confounding and reverse causation remain possible. Longitudinal or pre- and poststudies would be required to better assess the causal impact of MHFA training.

Second, although the invitation was distributed to the entire recruitment frame of French civil servants who had agreed to receive email communications, the low participation rate (1.93%) raises concerns about selection bias, increasing the risk of self-selection and limiting population representativeness. This low participation rate is consistent with voluntary nationwide web-based surveys conducted without incentives and may also reflect the sensitive nature of mental health as a survey topic. Individuals who chose to participate may have differed systematically from nonparticipants with respect to mental health interest, literacy, previous training experiences, or personal exposure to psychological distress. Consequently, the sample may not fully represent the overall French civil servant population.

In addition, participation in MHFA training itself is voluntary. Individuals who attend such training are likely to differ from nontrained individuals in motivation, professional interest, empathy, or preexisting mental health knowledge. Therefore, the higher KAB scores observed among MHFA-trained participants cannot be interpreted as evidence of a causal training effect and may partly reflect self-selection. Although multivariable analyses adjusted for age, gender, educational level, and professional category, residual confounding due to unmeasured characteristics remains possible. Regarding incomplete questionnaires, although some statistically significant differences were observed between complete and incomplete respondents, the corresponding effect sizes were negligible to small (Multimedia Appendix 5), suggesting limited selection based on the observed baseline characteristics. Only a limited set of baseline variables was available for incomplete respondents, preventing the development of a robust response-propensity model. Consequently, inverse probability weighting would have been limited in its ability to account for potentially outcome-related noncompletion and would itself have required substantial assumptions about the missingness mechanism. However, because KAB outcomes were unavailable for participants who did not complete the questionnaire, we cannot exclude the possibility that complete-case estimates remain affected by outcome-related bias. Therefore, the associations should be interpreted as observed within a large volunteer sample rather than estimates that can be directly generalized to all French civil servants.

Third, the questionnaire was adapted from a previously developed instrument but was not formally psychometrically validated in this population. Consequently, the KAB scores should be interpreted as descriptive indicators of mental health–related knowledge, attitudes, and behaviors rather than as measurements derived from a validated psychometric scale.

Fourth, the study population consisted exclusively of French civil servants affiliated with a specific health insurance and prevention system and who had agreed to receive email communications. Consequently, the findings may not be directly generalizable to other occupational groups, private-sector employees, or populations from different health care, organizational, or cultural contexts.

Fifth, the use of self-reported data could introduce social desirability bias, particularly regarding attitudes or prosocial behaviors. The anonymity of the survey and validation of the questionnaire mitigate this risk, but it cannot be excluded.

Sixth, time since training was used as an indirect proxy for retention and did not capture frequency of skill application, refresher training, workplace support, or other factors that may influence the long-term maintenance of MHFA-related competencies.

Despite these limitations, this study provides valuable descriptive data on mental health literacy and supportive behaviors among civil servants and offers a relevant baseline for future longitudinal and intervention-based research.

### Conclusions

This nationwide cross-sectional study highlights substantial heterogeneity in KAB related to psychological distress among French civil servants, with overall KAB levels remaining in the marginal range. Exposure to MHFA training was consistently associated with higher KAB scores, supporting its role as a public health literacy intervention in occupational settings. However, the observed attenuation of scores over time underscores the need to consider mental health competencies as a potentially declining dynamic rather than permanently acquired. Taken together, these findings support the relevance of integrating MHFA within broader and sustained prevention strategies, combining initial training with regular reinforcement mechanisms to strengthen mental health literacy. Their deployment in the civil service, and other workplace settings, would improve the collective capacity to respond to psychological distress, leading to greater social solidarity.

## Appendix

Multimedia Appendix 1 CROSS checklist.

Multimedia Appendix 2 Original French questionnaire.

Multimedia Appendix 3 English translation of the original French questionnaire.

Multimedia Appendix 4 Raw score ranges, normalization procedure, and normalized score ranges for the knowledge, attitudes, and behaviors dimensions.

Multimedia Appendix 5 Comparison between respondents who completed the entire questionnaire (N=4679) and those who completed only part of it but who at least provided their sociodemographic data (n=670).

Multimedia Appendix 6 Distribution of incomplete questionnaires according to the last completed questionnaire item.

Multimedia Appendix 7 Cohen *d* effect sizes for the comparison between Mental Health First Aid–trained and untrained participants.

Multimedia Appendix 8 Post-hoc power calculations for subgroup analyses according to time since Mental Health First Aid training.

## Abbreviations

ALGEE: Approach, Listen, Give support, Encourage professional help, Encourage other supports
CROSS: Checklist for Reporting Of Survey Studies
KAB: knowledge, attitudes, and behaviors
KAP: knowledge, attitudes, and practices
MHFA: Mental Health First Aid
WHO: World Health Organization

## Data Availability

The datasets generated or analyzed during this study are available from the corresponding author on reasonable request.
