# Supplementary material for: Assessment of Knowledge, Attitudes, and Behaviors Toward Others’ Mental Disorders to Improve Mental Health First Aid: Cross-Sectional Study in a French Workplace
Source: JMIR Public Health Surveill. 2026 Aug 21;12:e93207. doi: 10.2196/93207 (PMC13545527; doi:10.2196/93207)
Supplement: Supplementary file 2 [file publichealth_v12i1e93207_app2.docx]

| N° de question | Question | Réponse(s) [points] |
| --- | --- | --- |
| *Partie 1 : Données socio-démographiques et participation à la formation PSSM* | | |
| 1 | Quel est votre âge ? | - Entre 18 et 29 ans  - Entre 30 et 39 ans  - Entre 40 et 49 ans  - Entre 50 et 59 ans  - Entre 60 et 69 ans |
| 2 | Quel est votre genre ? | - Femme  - Homme  - Autre(s) |
| 3 | Quel est votre catégorie statuaire de la fonction publique ? | - Catégorie A  - Catégorie B  - Catégorie C |
| 4 | Quel était votre niveau d’étude ? | - Sans diplôme  - Inférieur au Bac (Brevet des Collèges, CAP ou BEP)  - Bac  - Bac + 2 (DUT, BTS, DEUG,…)  - Diplôme du 2^nd^ degré (licence, maîtrise, master 1...)  - Diplôme du 3^ème^ degré (master 2, DEA, DESS, Doctorat...) |
| 5 | Aviez-vous déjà été formé.e au domaine de la santé mentale ? | OUI  NON / Je ne sais pas |
| 6 | Avez-vous participé à une formation PSSM ? | - Oui <6 mois  - Oui 6 mois à 1 an  - Oui >1 an  - NON |
| 7 (Si Q6=Oui) | Quelles étaient vos principales motivations à participer à cette formation ? | - Pouvoir aider des personnes de mon entourage personnel (famille et/ou amis)  - En lien et utilité avec ma formation et/ou profession  - Pour moi-même, pour mieux comprendre ma propre santé mentale  - Par curiosité car le domaine de la santé mentale m’intéresse  - Afin de réaliser un acte citoyen et d’agir dans l’intérêt collectif  - Répondre à un besoin en santé mentale suite à la crise COVID  - Parce que cette formation est gratuite  - Autre |
| *Partie 2 : Connaissances* | | |
| Indiquez pour chacune des affirmations suivantes si elles vous semblent vraies ou fausses, en évitant le plus possible la réponse « je ne sais pas ». Les termes « maladies mentales » ou « troubles psychiques » englobant toute forme de mal-être psychique qui nécessite la prise d’un traitement ou le suivi d’une thérapie (dépression, bipolarité, troubles de la personnalité, etc.). | | |
| 8 | Certains troubles psychiques sont héréditaires. | - Vrai [1]  - Faux [0]  - Je ne sais pas [0] |
| 9 | Les personnes atteintes d’une maladie mentale pourraient s’en sortir si elles le voulaient vraiment. | - Vrai [0]  - Faux [1]  - Je ne sais pas [0] |
| 10 | Une maladie mentale n’est pas une vraie maladie d’un point de vue médical. | - Vrai [0]  - Faux [1]  - Je ne sais pas [0] |
| 11 | Les personnes atteintes d’une maladie mentale peuvent être un danger pour eux et pour les autres. | - Vrai [1]  - Faux [0]  - Je ne sais pas [0] |
| 12 | Je crois que le traitement d’une maladie mentale, prescrit par un professionnel de la santé mentale, ne serait pas efficace. | - Vrai [0]  - Faux [1]  - Je ne sais pas [0] |
| 13 | Une personne atteinte de schizophrénie peut se rétablir sans médicaments. | - Vrai [0]  - Faux [1]  - Je ne sais pas [0] |
| 14 | Les personnes qui présentent un trouble anxieux peuvent être confrontées aux situations anxiogènes pour se rétablir. | - Vrai [1]  - Faux [0]  - Je ne sais pas [0] |
| Pour chacun des acteurs mentionnés ci-dessous, répondez par oui ou non si vous pensez qu’ils peuvent réaliser les actions suivantes : | | |
| 15 | Reconnaître une personne en difficulté psychique et apporter une aide en première intention ? | - Un.e formé(e) aux PSSM  🡪 OUI : [1]  🡪 NON : [0]  - Un.e psychologue  🡪 OUI : [1]  🡪 NON : [0]  - Un.e psychiatre  🡪 OUI : [1]  🡪 NON : [0] |
| 16 | Poser le diagnostic d’un trouble psychique ? | - Un.e formé(e) aux PSSM  🡪 OUI : [0]  🡪 NON : [1]  - Un.e psychologue  🡪 OUI : [1]  🡪 NON : [0]  - Un.e psychiatre  🡪 OUI : [1]  🡪 NON : [0] |
| 17 | Réaliser une psychothérapie ? | - Un.e formé(e) aux PSSM  🡪 OUI : [0]  🡪 NON : [1]  - Un.e psychologue  🡪 OUI : [1]  🡪 NON : [0]  - Un.e psychiatre  🡪 OUI : [1]  🡪 NON : [0] |
| *Partie 3 : Attitudes* | | |
| 18 | Seriez-vous prêt.e à devenir ami avec une personne atteinte d’une maladie mentale ? | - Vrai [1]  - Faux [0]  - Je ne sais pas [0] |
| 19 | Seriez-vous prêt.e à travailler en étroite collaboration avec une personne atteinte d'une maladie mentale ? | - Vrai [1]  - Faux [0]  - Je ne sais pas [0] |
| 20 | Généralement, une personne présentant un trouble psychique peut participer aux décisions concernant sa santé. | - Vrai [1]  - Faux [0]  - Je ne sais pas [0] |
| 21 | Si une personne fait une attaque de panique, je n'interviens pas, car elle finira par se calmer. | - Vrai [0]  - Faux [1]  - Je ne sais pas [0] |
| *Partie 4 : Pratiques* | | |
| 22 | Vous ne devriez jamais demander à une personne si elle pense au suicide, car cela pourrait lui en donner l'idée. | - Vrai [0]  - Faux [1]  - Je ne sais pas [0] |
| 23 | Quand vous assistez une personne touchée par un trouble psychotique, il est préférable de lui demander ce qui pourrait l’aider pour la laisser choisir et augmenter son sentiment de sécurité, tout en restant calme et en utilisant des phrases courtes et claires. | - Vrai [1]  - Faux [0]  - Je ne sais pas [0] |
| 24 | Si votre ami a un trouble dépressif, il devra suivre un traitement médicamenteux. | - Vrai [0]  - Faux [1]  - Je ne sais pas [0] |
| 25 | Si votre ami a un trouble dépressif, vous pouvez lui conseiller de suivre une thérapie. | - Vrai [1]  - Faux [0]  - Je ne sais pas [0] |
| 26 | Une personne qui fait une crise psychotique pourrait être contrainte à être hospitalisée. | - Vrai [1]  - Faux [0]  - Je ne sais pas [0] |
| 27 (Si Q6=Oui) | Pour chacune de propositions suivantes, dites si elle fait partie du plan d'action AÉRER ou non | - Essayer de soigner la personne ou de trouver des réponses à ses problèmes  - Assister et évaluer en cas de crise  - Réconforter et informer  - Écouter activement et sans jugement  - Encourager à se ressaisir |
| 28 (Si Q6=Oui) | Avez-vous déjà appliqué vos compétences de Premiers Secours en Santé Mentale afin de venir en aide à une ou des personne(s) ayant un problème de santé mentale ? | - Oui  - Non |
| 29 (Si Q28=Oui) | Auprès de combien de personne(s) environ ? | - 1  - Entre 2 et 4  - Entre 5 et 7  - Entre 8 et 10  - Plus de 10 |
| 30 (Si Q28=Oui) | Diriez-vous que les notions PSSM acquises ont permis d’aider cette ou ces personnes ? | - Oui  - Non  - Je ne sais pas |
| Estimez votre niveau de confiance pour chacun des items suivants (0 : pas du tout en confiance - 4 : totalement en confiance) : | | |
| 31 | Reconnaître les signes du développement d’un trouble psychique ou d’une crise en santé mentale | 0 1 2 3 4 [0-4] |
| 32 | Approcher, évaluer et assister quelqu’un qui est en train de développer un trouble psychique ou une crise en santé mentale | 0 1 2 3 4 [0-4] |
| 33 | Demander à une personne si elle a des pensées suicidaires | 0 1 2 3 4 [0-4] |
| 34 | Informer une personne et lui offrir du soutien (réconforter) par rapport à ses problèmes de santé mentale | 0 1 2 3 4 [0-4] |
| 35 | Écouter et interagir avec une personne, sans exprimer un jugement sur la situation | 0 1 2 3 4 [0-4] |
| 36 | Encourager une personne à chercher de l’aide professionnelle appropriée | 0 1 2 3 4 [0-4] |
| 37 | Encourager une personne à chercher d’autres soutien et renseigner sur les autres ressources disponibles | 0 1 2 3 4 [0-4] |
| 38 | Reconnaître et corriger les mécompréhensions d’autres personnes au sujet des troubles de santé mentale | 0 1 2 3 4 [0-4] |
