# Supplementary material for: Assessment of Knowledge, Attitudes, and Behaviors Toward Others’ Mental Disorders to Improve Mental Health First Aid: Cross-Sectional Study in a French Workplace
Source: JMIR Public Health Surveill. 2026 Aug 21;12:e93207. doi: 10.2196/93207 (PMC13545527; doi:10.2196/93207)
Supplement: Supplementary file 3 [file publichealth_v12i1e93207_app3.docx]

Note. This is an English translation of the original French questionnaire. Scoring is unchanged.

| Question No. | Question | Response(s) [score] |
| --- | --- | --- |
| *Part 1: Sociodemographic characteristics and MHFA training status* | | |
| 1 | What is your age? | - 18–29 years - 30–39 years - 40–49 years - 50–59 years - 60–69 years |
| 2 | What is your gender? | - Woman  - Man  - Other |
| 3 | What is your public service employment category? | - Category A  - Category B  - Category C |
| 4 | What is your highest educational level? | - No diploma  - Below high school diploma  - High school diploma (French Baccalaureate)  - First-cycle university degree (Baccalaureate + 2 years)  - Second-cycle university degree (Baccalaureate + 3 years)  - Third-cycle university degree (Baccalaureate + 5 years and more) |
| 5 | Have you previously received training in mental health? | Yes  No / I don’t know |
| 6 | Have you attended a Mental Health First Aid (MHFA) training course? | Yes, <6 months ago  Yes, 6 months–1 year ago  Yes, >1 year ago  No |
| 7 (If Q6=Yes) | What were your main motivations for attending this training? | - To better support family members or friends - Related to and useful for my studies and/or profession - To improve my understanding of my own mental health - Due to my interest in the field of mental health - To engage in a civic action and act in the public interest - To address a mental health need arising from the COVID crisis - Because this training was free - Other |
| *Part 2 : Knowledge* | | |
| For each statement below, indicate whether you think it is true or false. Avoid answering 'I don't know' whenever possible. The terms 'mental illness' or 'mental disorders' refer to any psychological condition requiring treatment or therapy. | | |
| 8 | Some mental disorders are hereditary. | - True [1]  - False [0]  - I don’t know [0] |
| 9 | People with mental illness could recover if they really wanted to. | - True [0]  - False [1]  - I don’t know [0] |
| 10 | Mental illness is not a real medical illness. | - True [0]  - False [1]  - I don’t know [0] |
| 11 | People with mental illness may be a danger to themselves or others. | - True [1]  - False [0]  - I don’t know [0] |
| 12 | I believe treatment prescribed by a mental health professional would not be effective. | - True [0]  - False [1]  - I don’t know [0] |
| 13 | A person with schizophrenia can recover without medication. | - True [0]  - False [1]  - I don’t know [0] |
| 14 | People with anxiety disorders can confront anxiety-provoking situations to recover. | - True [1]  - False [0]  - I don’t know [0] |
| For each of the actors mentioned below, answer yes or no as to whether you think they can carry out the following actions: | | |
| 15 | Who can recognize a person experiencing mental health difficulties and provide initial support? | - MHFA-trained person  🡪 Yes : [1]  🡪 No : [0]  - Psychologist  🡪 Yes : [1]  🡪 No : [0]  - Psychiatrist  🡪 Yes : [1]  🡪 No : [0] |
| 16 | Who can diagnose a mental disorder? | - MHFA-trained person  🡪 Yes : [0]  🡪 No : [1]  - Psychologist  🡪 Yes : [1]  🡪 No : [0]  - Psychiatrist  🡪 Yes : [1]  🡪 No : [0] |
| 17 | Who can provide psychotherapy? | - MHFA-trained person  🡪 Yes : [0]  🡪 No : [1]  - Psychologist  🡪 Yes : [1]  🡪 No : [0]  - Psychiatrist  🡪 Yes : [1]  🡪 No : [0] |
| *Part 3 : Attitudes* | | |
| 18 | Would you be willing to become friends with a person with mental illness? | - Yes [1]  - No [0]  - I don’t know [0] |
| 19 | Would you be willing to work closely with a person with mental illness? | - Yes [1]  - No [0]  - I don’t know [0] |
| 20 | Generally, a person with a mental disorder can participate in decisions regarding their healthcare. | - Yes [1]  - No [0]  - I don’t know [0] |
| 21 | If someone is having a panic attack, I would not intervene because they will eventually calm down. | - Yes [0]  - No [1]  - I don’t know [0] |
| *Part 4 : Behaviors* | | |
| 22 | You should never ask someone whether they are thinking about suicide because it might give them the idea. | - True [0]  - False [1]  - I don’t know [0] |
| 23 | When assisting someone experiencing psychosis, it is preferable to ask what might help them, remain calm, and use short, clear sentences. | - True [1]  - False [0]  - I don’t know [0] |
| 24 | If your friend has depression, they will need medication. | - True [0]  - False [1]  - I don’t know [0] |
| 25 | If your friend has depression, you can advise them to seek therapy. | - True [1]  - False [0]  - I don’t know [0] |
| 26 | A person experiencing a psychotic episode may need to be hospitalized. | - True [1]  - False [0]  - I don’t know [0] |
| 27 (if Q6=Yes) | For each statement below, indicate whether it is part of the MHFA ALGEE action plan. | - Try to solve the person's problems - Assist and assess in a crisis - Give reassurance and information - Listen non-judgmentally - Encourage the person to 'pull themselves together' |
| 28 (if Q6=Yes) | Have you ever used your MHFA skills to help someone experiencing a mental health problem? | - Yes  - No |
| 29 (if Q28=Yes) | Approximately how many people have you helped? | 1  2–4  5–7  8–10  More than 10 |
| 30 (if Q28=Yes) | Do you think the MHFA knowledge acquired helped these individuals? | - Yes  - No  - I don’t know |
| Rate your confidence for each of the following skills (0 = not at all confident; 4 = completely confident): | | |
| 31 | Recognize signs of a developing mental disorder or mental health crisis | 0 1 2 3 4 [0-4] |
| 32 | Approach, assess and assist someone developing a mental disorder or mental health crisis | 0 1 2 3 4 [0-4] |
| 33 | Ask someone whether they have suicidal thoughts | 0 1 2 3 4 [0-4] |
| 34 | Provide information and reassurance | 0 1 2 3 4 [0-4] |
| 35 | Listen without judgment | 0 1 2 3 4 [0-4] |
| 36 | Encourage appropriate professional help | 0 1 2 3 4 [0-4] |
| 37 | Encourage other sources of support | 0 1 2 3 4 [0-4] |
| 38 | Recognize and correct misconceptions about mental health | 0 1 2 3 4 [0-4] |
