# Supplementary material for: Assessment of Knowledge, Attitudes, and Behaviors Toward Others’ Mental Disorders to Improve Mental Health First Aid: Cross-Sectional Study in a French Workplace
Source: JMIR Public Health Surveill. 2026 Aug 21;12:e93207. doi: 10.2196/93207 (PMC13545527; doi:10.2196/93207)
Supplement: Supplementary file 4 [file publichealth_v12i1e93207_app4.docx]

**Table S1.** Raw score ranges, normalization procedure, and normalized score ranges for the Knowledge, Attitudes, and Behaviors (KAB) dimensions.

| **Dimension** | **Number of items** | **Raw score range** | **Maximum possible score** | **Normalized score range** |
| --- | --- | --- | --- | --- |
| Knowledge | 16 | 0–16 | 16 | 0–40 |
| Attitudes | 4 | 0–4 | 4 | 0–40 |
| Behaviors | 17 | 0–37 | 37 | 0–40 |
| Total KAB score | - | - | - | 0–120 |
