# Supplementary material for: Assessment of Knowledge, Attitudes, and Behaviors Toward Others’ Mental Disorders to Improve Mental Health First Aid: Cross-Sectional Study in a French Workplace
Source: JMIR Public Health Surveill. 2026 Aug 21;12:e93207. doi: 10.2196/93207 (PMC13545527; doi:10.2196/93207)
Supplement: Supplementary file 5 [file publichealth_v12i1e93207_app5.docx]

**Table S1.** Comparison between respondents who completed the entire questionnaire (n = 4,679) and those who completed only part of it but who at least provided their sociodemographic data (n = 670).

| **Variable** | **Respondents who completed the entire questionnaire (n=4679)** | **Respondents who completed only part of the questionnaire (n=670)** | **p-value** | **Cramér's V** |
| --- | --- | --- | --- | --- |
| Age 18–29 | 59/4679 (1.26%) | 14/670 | <0.001 | 0.078 (negligible) |
| Age 30–39 | 303/4679 (6.48%) | 48/670 |  |  |
| Age 40–49 | 1075/4679 (22.97%) | 110/670 |  |  |
| Age 50–59 | 1924/4679 (41.12%) | 250/670 |  |  |
| Age 60–69 | 1318/4679 (28.17%) | 248/670 |  |  |
| Woman | 3022/4679 (64.59%) | 373/670 | <0.001 | 0.062 (negligible) |
| Man | 1650/4679 (35.26%) | 295/670 |  |  |
| Other | 7/4679 (0.15%) | 2/670 |  |  |
| Category A | 2207/4679 (47.17%) | 250/670 | <0.001 | 0.069 (negligible) |
| Category B | 1777/4679 (37.98%) | 287/670 |  |  |
| Category C | 695/4679 (14.85%) | 133/670 |  |  |
| No diploma | 39/4679 (0.83%) | 10/670 | <0.001 | 0.110 (negligible to small) |
| Below high school diploma | 433/4679 (9.25%) | 111/670 |  |  |
| High school | 959/4679 (20.50%) | 162/670 |  |  |
| First-cycle university degree | 878/4679 (18.76%) | 143/670 |  |  |
| Second-cycle university degree | 1342/4679 (26.68%) | 147/670 |  |  |
| Third-cycle university degree | 1028/4679 (21.97%) | 97/670 |  |  |
| Previous mental health training | 1027/4679 (21.95%) | 132/670 | 0.204 | 0.017 (no nifference) |
| No previous mental health training or do not know | 3652/4679 (78.05%) | 538/670 |  |  |
| Previous MHFA training <6 months ago | 216/4679 (4.62%) | 33/670 | 0.749 | 0.017 (no difference) |
| Previous MHFA training 6-12 months ago | 117/4679 (2.50%) | 18/670 |  |  |
| Previous MHFA training >12 months ago | 212/4679 (4.53%) | 36/670 |  |  |
| No previous MHFA training | 4134/4679 (88.35%) | 583/670 |  |  |
