# Supplementary material for: Assessment of Knowledge, Attitudes, and Behaviors Toward Others’ Mental Disorders to Improve Mental Health First Aid: Cross-Sectional Study in a French Workplace
Source: JMIR Public Health Surveill. 2026 Aug 21;12:e93207. doi: 10.2196/93207 (PMC13545527; doi:10.2196/93207)
Supplement: Supplementary file 6 [file publichealth_v12i1e93207_app6.docx]

**Table S1.** Distribution of incomplete questionnaires according to the last completed questionnaire item.

| **Last completed questionnaire item** | **Questionnaire section** | **Participants, n** | **% of incomplete questionnaires** |
| --- | --- | --- | --- |
| Q1 | Age | 670 | 100 |
| Q2 | Gender | 670 | 100 |
| Q3 | Professional category | 670 | 100 |
| Q4 | Educational level | 670 | 100 |
| Q5 | Previous mental health training | 670 | 100 |
| Q6 | Previous MHFA training | 670 | 100 |
| Q7 | Motivation for MHFA training | 670 | 100 |
| Q8 | Knowledge | 230 | 34 |
| Q9 | Knowledge | 226 | 34 |
| Q10 | Knowledge | 226 | 34 |
| Q11 | Knowledge | 226 | 34 |
| Q12 | Knowledge | 225 | 34 |
| Q13 | Knowledge | 225 | 34 |
| Q14 | Knowledge | 221 | 33 |
| Q15 | Knowledge | 214 | 32 |
| Q16 | Knowledge | 214 | 32 |
| Q17 | Knowledge | 212 | 32 |
| Q18 | Attitudes | 178 | 27 |
| Q19 | Attitudes | 178 | 27 |
| Q20 | Attitudes | 178 | 27 |
| Q21 | Attitudes | 178 | 27 |
| Q22 | Behaviors | 4 | 1 |
| Q23 | Behaviors | 3 | <1 |
| Q24 | Behaviors | 4 | 1 |
| Q25 | Behaviors | 4 | 1 |
| Q26 | Behaviors | 4 | 1 |
| Q27 | Behaviors | 2 | <1 |
| Q28 | Behaviors | 2 | <1 |
| Q29 | Behaviors | 1 | <1 |
| Q30 | Behaviors | 1 | <1 |
| Q31 | Behaviors | 1 | <1 |
| Q32 | Behaviors | 3 | <1 |
| Q33 | Behaviors | 2 | <1 |
| Q34 | Behaviors | 2 | <1 |
| Q35 | Behaviors | 2 | <1 |
| Q36 | Behaviors | 2 | <1 |
| Q37 | Behaviors | 2 | <1 |
| Q38 | Behaviors | 2 | <1 |
| **Total** |  | **670** | **100** |
