# Supplementary material for: Assessment of Knowledge, Attitudes, and Behaviors Toward Others’ Mental Disorders to Improve Mental Health First Aid: Cross-Sectional Study in a French Workplace
Source: JMIR Public Health Surveill. 2026 Aug 21;12:e93207. doi: 10.2196/93207 (PMC13545527; doi:10.2196/93207)
Supplement: Supplementary file 7 [file publichealth_v12i1e93207_app7.docx]

**Table S1.** Cohen's *d* effect sizes for the comparison between MHFA-trained and untrained participants

| **Outcome** | **Untrained Mean (SD)** | **Trained Mean (SD)** | **Mean difference** | **Cohen's d** | **Interpretation** |
| --- | --- | --- | --- | --- | --- |
| **Knowledge** | 31.04 (5.35) | 33.31 (3.96) | 2.27 | **0.44** | Small-to-moderate |
| **Attitude** | 29.10 (11.70) | 34.35 (8.81) | 5.25 | **0.46** | Small-to-moderate |
| **Behavior** | 21.06 (7.84) | 27.87 (6.13) | 6.81 | **0.89** | Large |
| **KAB** | 81.21 (19.09) | 95.56 (14.17) | 14.35 | **0.77** | Moderate-to-large |
