# Supplementary material for: Assessment of Knowledge, Attitudes, and Behaviors Toward Others’ Mental Disorders to Improve Mental Health First Aid: Cross-Sectional Study in a French Workplace
Source: JMIR Public Health Surveill. 2026 Aug 21;12:e93207. doi: 10.2196/93207 (PMC13545527; doi:10.2196/93207)
Supplement: Supplementary file 8 [file publichealth_v12i1e93207_app8.docx]

**Table S1.** Post-hoc power calculations for subgroup analyses according to time since MHFA training.

| **Outcome** | **Cohen's** **f** | **Achieved statistical power (%)** |
| --- | --- | --- |
| Knowledge score | 0.189 | 98.3 |
| Attitude score | 0.089 | 44.4 |
| Behavior score | 0.159 | 92.1 |
| Total KAB score | 0.174 | 96.1 |

Note. Achieved statistical power was estimated for the predefined one-way ANOVA subgroup analyses using the observed effect size (Cohen's *f*), the observed subgroup sample sizes (n = 216, 117, and 212), and a two-sided significance level of 0.05.
